# Modified Two-Stage Approach for Management of Combined Rhegmatogenous Retinal Detachment and Choroidal Detachment With Extreme Hypotony

**DOI:** 10.7759/cureus.38653

**Published:** 2023-05-06

**Authors:** Vinita Gupta, Sandhya Makhija, Neelam Khatwani, Saurabh Luthra

**Affiliations:** 1 Ophthalmology, All India Institute of Medical Sciences, Rishikesh, Rishikesh, IND; 2 Ophthalmology, Sant Parmanand Hospital, Delhi, IND; 3 Ophthalmology, Drishti Eye Institute, Dehradun, IND

**Keywords:** drainage of suprachoroidal fluid, steroids, pars plana vitrectomy, hypotony, rhegmatogenous retinal detachment with choroidal detachment

## Abstract

Combined rhegmatogenous retinal detachment (RRD) and serous choroidal detachment (CD) present a significant challenge. No global standard of care exists for treating these complex RRDs. There is a lower failure rate when such detachments are treated with pars plana vitrectomy than with scleral buckle alone. The use of pre-operative steroids may not work in cases with moderate-to-severe CDs with severe hypotony where suprachoroidal fluid drainage is required to reduce inflammatory mediators, thus preventing proliferative vitreoretinopathy (PVR). We report a case of a 62-year-old male who had a combined RRD and severe CD with vitreous hemorrhage in the left eye (LE). There was extreme hypotony leading to a severely deformed and distorted globe with poor visualization of the fundus. The patient was started on 60 mg of oral prednisolone, and a posterior subtenon injection of 20 mg of triamcinolone acetonide was given to reduce inflammation and CD. However, despite one week of pre-operative steroids, there was severe hypotony. The patient was taken for pars plana vitrectomy with drainage of suprachoroidal fluid. Intra-operatively even after drainage of suprachoroidal fluid via inferotemporal posterior sclerotomy, hypotony persisted, and media was very hazy, precluding us from proceeding with vitrectomy in the first sitting. Oral steroids were continued, and vitrectomy was done in the second sitting, 72 hours later, with long-term silicone oil tamponade. Post-operatively patient had a well-formed globe with an attached retina and a good visual acuity. Our case thereby highlights that combined retinal and CD is a complicated diagnosis that presents with many pre-operative, intra-operative, and post-operative challenges. We could achieve good anatomical and functional success using a modified two-stage approach in our unusual case of combined RRD wth CD with extreme hypotony.

## Introduction

Rhegmatogenous retinal detachment (RRD) with choroidal detachment (CD) is a special subset of RRD with rapid progression and a high failure rate occurring in only 2-8.6% of all RRD cases [1]. It is usually associated with hypotony, anterior, and posterior uveitis, and higher chances of proliferative vitreoretinopathy (PVR). It occurs with increased frequency in high myopes, people with aphakia and pseudophakia, the elderly, and those with a history of ocular trauma [2]. These cases of combined RRD with CD show poor results with conventional buckling and show better surgical outcomes with pars plana vitrectomy [1-3]. Although the treatment success rate of RRD with CD has improved with the introduction of vitrectomy and the use of perioperative pharmacological management [4-7], there is no consensus on the best approach for these patients of combined RRD with CD who have severe hypotony. Herein we describe our experience of surgical intervention with good outcome in an unusual presentation of RRD with CD, which was associated with extreme hypotony.

## Case presentation

**Figure 1:**
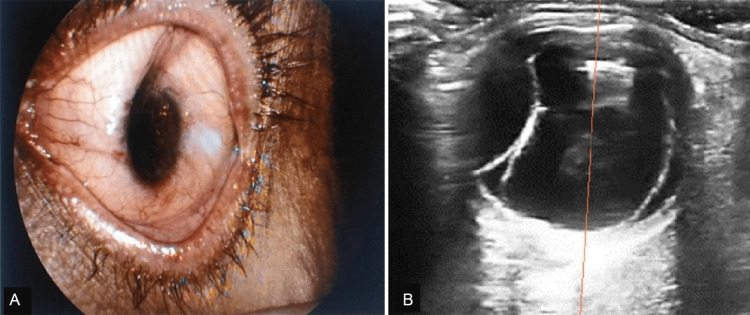
Preoperative photographs of the left eye at presentation. (A) Slit lamp biomicroscopic photograph showing a deformed globe, corneal edema, and flat anterior chamber. (B) Ultrasound B-scan image showing vitreous hemorrhage with retinal detachment and choroidal detachment.

A 62-year-old male, pseudophakic in both eyes, presented to our ED with sudden painless vision loss in the left eye (LE) for one week. He was diagnosed elsewhere as a case of LE vitreous hemorrhage and was put on conservative treatment. Ocular examination revealed a best corrected visual acuity (BCVA) of hand movements close to the face in the LE with a severely deformed and distorted globe with corneal edema, a flat anterior chamber, non-dilating pupil, pseudophakia, and unrecordably low intraocular pressure (IOP) (Figure 1A). There was poor visualization of the fundus because of the associated vitreous hemorrhage. The right eye had BCVA of 6/6 with IOP of 14 mm Hg with pseudophakia with a normal fundus examination. A-scan ultrasonography of both eyes revealed normal axial length in both eyes. In contrast, B-scan ultrasonography of LE showed hyper-reflective mobile dot-like echoes within the vitreous cavity suggestive of vitreous hemorrhage, along with a highly reflective membranous echo with limited after movements, attached at ora serrata anteriorly and optic disc posteriorly suggestive of a retinal detachment. Also, there was a smooth, dome-shaped, highly reflective membranous echo with no after-movements extending from the ciliary body region anteriorly and attached distally to the optic nerve posteriorly, suggestive of choroidal detachment (Figure 1B).

**Figure 2:**
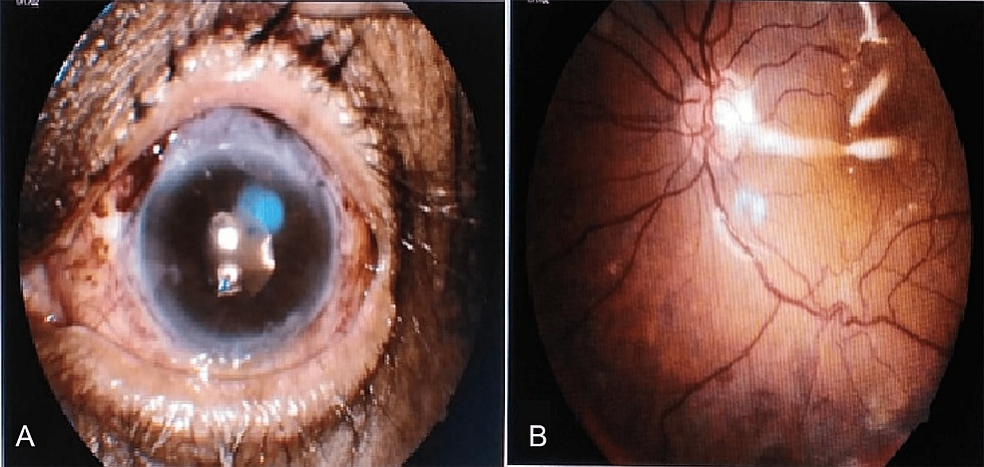
Photographs of the left eye in the early postoperative period. (A) Slit lamp biomicroscopic photograph showing a well-formed globe with a clear cornea and a posterior chamber intraocular lens in situ. (B) Fundus photograph showing an attached retina under silicone oil.

Based on the above findings, the patient was diagnosed with LE combined RRD and CD with associated vitreous hemorrhage and extreme hypotony and was planned for pars plana vitrectomy with silicone oil tamponade. He was started on 60 mg of oral prednisolone, histamine H2 receptor antagonists, topical prednisolone acetate 1% six times a day, and 1% atropine eye ointment three times a day in the LE. The patient was also given a posterior subtenon injection of triamcinolone acetonide 20 mg using a 25-gauge needle bevel down, taking extreme care so as not to cause any globe perforation in such an eye with extreme hypotony. The patient was reviewed every two days. At one week, IOP in LE was still non-recordable, and an ultrasound B scan showed the persistence of significant CD. Hence, the patient was taken for 25-gauge pars plana vitrectomy. However, intra-operatively due to significant hypotony and globe distortion, insertion of trocar cannulas was difficult. Thus, to drain suprachoroidal fluid, posterior sclerotomy was done inferotemporally, and to facilitate this drainage, an anterior chamber maintainer and viscoelastic agent were used to deepen the anterior chamber and pressurize the eye. However, hypotony persisted, and the media was very hazy, precluding us from proceeding with vitrectomy. Therefore, the sclerotomy site was sutured, oral steroids continued, and the patient was planned for vitrectomy in the second sitting. After another 72 hours, IOP had built up at review, and the globe contour had been restored. A repeat ultrasound B-scan at this time showed a significant decrease in CD. 25-gauge pars plana vitrectomy was hence performed. Intra-operatively multiple U-shaped tears were found in the superotemporal and temporal retinal periphery, along with grade C PVR. After the air-fluid exchange, endolaser to retinal breaks and silicone oil tamponade were done. The patient had a good anatomical outcome in the early postoperative period with a formed anterior chamber and an attached retina under silicone oil (Figure 2). There was an improvement in the BCVA to 6/60. At a six-month follow-up, the patient maintained an attached retina in the LE with a normal IOP and had a BCVA of 6/18.

## Discussion

RRD with CD is a special subset of RRD with rapid progression and a higher failure rate, resulting in poor anatomical and functional success. Although the exact pathogenic mechanism of CD following RRD is not clearly established, most investigators believe that hypotony induced by retinal detachment stimulates dilatation and hyperpermeability of choroidal vessels leading to CD. This is further compounded by ciliary body edema and detachment, which reduces the aqueous humor production and outflow, increasing the supra choroidal effusion and hypotony. Therefore, a positive feedback mechanism is established. This is associated with the breakdown of the blood-ocular barrier, inflammatory mediators release, retinal pigment epithelium migration, and PVR development [8]. Pars plana vitrectomy (PPV) is advocated for the primary treatment of combined RRD with CD. This results in improved success rates, as all retinal breaks can be identified, and complete excision of proliferative and concentrated vitreous is possible with PPV [5,7,9]. Severe CD may persist for some patients at the time of PPV despite preoperative administration of steroids, as in our case, who had persistence of significant CD and hypotony. Retinal apposition in these cases poses a unique surgical challenge. Also, performing a vitrectomy in a hypotonic eye with CD is technically difficult. A more extensive posterior vitrectomy in conjunction with drainage of choroidal effusion is necessary for these eyes [5,10]. Though it is the era of transconjunctival micro-incision vitrectomy surgery, a 20-gauge sclerotomy may be favorable for CD drainage before PPV, as we did in our case [11]. Inserting an infusion cannula in such eyes with severe hypotony, hazy media, and suprachoroidal effusion, in our experience, is another intraoperative challenge. Yang CM [12] has shown that inserting a 6-mm-long infusion cannula into the vitreous cavity may be useful for normal RRD with CD. We also tried inserting a 6-mm long cannula; however, insertion was not easy due to extremely low IOP. As the edge of the trocar could have increased the risk of damage to the pars plana in such a hypotonic eye with the possibility of suprachoroidal hemorrhage, vitrectomy was deferred to a later date. Since the risk of postoperative hypotony is high in these patients, silicone oil endo tamponade has been advocated for these patients [13]. We also used silicone oil to ensure prolonged postoperative tamponade because of the high potential for re-detachment.

## Conclusions

Our case thereby highlights that combined RRD with CD is a complicated diagnosis that presents with many preoperative, intra-operative, and postoperative challenges. Pars plana vitrectomy is beneficial as it allows control of IOP preoperatively, facilitating early drainage of suprachoroidal fluid via the sclerotomies. This obviates the need to wait for steroid-induced resolution of CD, which is usually prolonged with the consequent increased risk of preoperative PVR. However, postoperative long-term risks of PVR need for silicone oil removal are issues that still need to be addressed. We could achieve good anatomical and functional success using the modified two-stage approach in an unusual presentation of combined RRD with CD with extreme hypotony.
